# *INHBA* is a Prognostic Biomarker and Correlated With Immune Cell Infiltration in Cervical Cancer

**DOI:** 10.3389/fgene.2021.705512

**Published:** 2022-01-04

**Authors:** Kaidi Zhao, Yuexiong Yi, Zhou Ma, Wei Zhang

**Affiliations:** Department of Obstetrics and Gynecology, Zhongnan Hospital of Wuhan University, Wuhan, China

**Keywords:** INHBA, biomarker, cervical cancer, prognosis, immune infiltration

## Abstract

**Background:** Inhibin A (*INHBA*), a member of the *TGF*-*β* superfamily, has been shown to be differentially expressed in various cancer types and is associated with prognosis. However, its role in cervical cancer remains unclear.

**Methods:** We aimed to demonstrate the relationship between *INHBA* expression and pan-cancer using The Cancer Genome Atlas (TCGA) database. Next, we validated *INHBA* expression in cervical cancer using the Gene Expression Omnibus (GEO) database, including GSE7803, GSE63514, and GSE9750 datasets. Enrichment analysis of *INHBA* was performed using the R package “clusterProfiler.” We analyzed the association between immune infiltration level and *INHBA* expression in cervical cancer using the single-sample gene set enrichment analysis (ssGSEA) method by the R package GSVA. We explored the association between *INHBA* expression and prognosis using the R package “survival”.

**Results:** Pan-cancer data analysis showed that *INHBA* expression was elevated in 19 tumor types, including cervical cancer. We further confirmed that *INHBA* expression was higher in cervical cancer samples from GEO database and cervical cancer cell lines than in normal cervical cells. Survival prognosis analysis indicated that higher *INHBA* expression was significantly associated with reduced Overall Survival (*p* = 0.001), disease Specific Survival (*p* = 0.006), and Progression Free Interval (*p* = 0.001) in cervical cancer and poorer prognosis in other tumors. GSEA and infiltration analysis showed that *INHBA* expression was significantly associated with tumor progression and some types of immune infiltrating cells.

**Conclusion:** *INHBA* was highly expressed in cervical cancer and was significantly associated with poor prognosis. Meanwhile, it was correlated with immune cell infiltration and could be used as a promising prognostic target for cervical cancer.

## 1 Introduction

Although the introduction of prophylactic vaccination against human papillomavirus (HPV) and screening would substantially reduce the incidence of cervical cancer, it remains the most common gynecologic malignancy worldwide Matsuo et al. (2017). The global mortality rate from cervical cancer is approximately 54% Kiran et al. (2018). In 2018, an estimated 570 000 cases of cervical cancer were diagnosed, and 311 000 women died from the disease Canfell et al. (2020). Persistent infection with high-risk types of HPV (hrHPV) is a necessary cause of cervical cancer; however, alterations in tumor-suppressor genes and/or oncogenes may also be necessary for cervical cancer progression Babion et al. (2018). The combination of early detection *via* screening and effective treatment with surgery, chemotherapy, and radiotherapy has meant that early stage cervical cancer can be successfully treated, with 5-years overall survival (OS) rates as high as 90% Potikanond et al. (2017). However, metastatic cervical cancer is virtually incurable, mainly due to limited treatment options, with 5-years OS rates below 10% Wallbillich et al. (2020). Thus, better prognostic biomarkers for cervical cancer development are urgently required to increase patient survival.

Inhibin A (*INHBA*), a member of the transforming growth factor-*β* superfamily, is located in 7p14.1, and encodes the *β*A-subunit of the activins/inhibins Tournier et al. (2014). In mammals, prolonged upregulation of *INHBA* has been associated with cardiac remodeling and failure Dogra et al. (2017). *INHBA* also plays a crucial role in follicle activation in females Bellingham et al. (2013) and encodes for the major regulator of FSH secretion in adult males d’Aurora et al. (2015). Previous studies have identified an association between *INHBA* expression and invasion or poor survival in cancer Singh et al. (2018); Lee et al. (2015); Seder et al. (2009); Chen et al. (2019); Okano et al. (2013); Antsiferova et al. (2017). The possible mechanism of INHBA contribution to tumorigenesis lies in its interaction with other receptor subunits or other molecular partners depending on their relative expression levels. Liu et al. found that *INHBA* expression level is associated with cancer aggressiveness and may be a potential diagnostic marker of invasive breast cancer Liu et al. (2018). Moreover, *INHBA* has been demonstrated to be a significantly mutated driver candidate gene in endometrial cancer Kadara et al. (2017). Although it has been proven that *INHBA* is important for tumor development, no study has explored the precise function and mechanism of *INHBA* in cervical cancer.

In this study, we comprehensively evaluated the difference in *INHBA* expression between normal and tumor tissues using RNA-seq data from The Cancer Genome Atlas (TCGA) database as well as its association with patient prognosis. Furthermore, to reveal its potential functions, we performed gene set enrichment analysis (GSEA) on the low and high expression groups of *INHBA*. Finally, we examined the correlation between *INHBA* expression and immune cell infiltration levels to explore the possible mechanism by which *INHBA* induces tumor occurrence and progression.

## 2 Materials and Methods

### 2.1 Collection of *INHBA* Expression Data From TCGA Database

*INHBA* expression data with clinical information (including 13 normal and 306 cervical cancer tissues) were obtained from TCGA public database (cancergenome.nih.gov). Level 3 HTSeq-fragments per kilobase per million (FPKM) samples were computed with an HTseq tool and subsequently transformed to transcripts per million (TPM) units. We also downloaded publicly available transcript data from TCGA and Genotype-Tissue Expression (GTEx) database, which was uniformly managed by the Toil process from UCSC Xena (https://xenabrowser.net/datapages/). Cervical cancer microarray data were obtained from the Gene Expression Omnibus (GEO) database, including GSE7803 (Platform: GPL96), GSE63514 (Platform: GPL570), and GSE9750 (Platform: GPL96) datasets.

### 2.2 qRT-PCR

We selected an RNAsimple Total RNA Kit (TIANGEN, Beijing, China) to extract total RNA from cervical cancer cell lines. The Servicebio®RT First Strand cDNA Synthesis Kit was used for qRT-PCR (Servicebio, Wuhan, China). Next, we performed real-time PCR using TB Green^®^ Premix Ex TaqTM (Takara, Japan). The primers used were as follows: human *INHBA* -Forward: 5′-ATC​ATC​ACG​TTT​GCC​GAG​TCA-3′; human *INHBA* -Reverse: 5′-GAA​GAG​GCG​GAT​GGT​GAC​TTT-3′; human GAPDH-Forward: 5′-GGA​GTC​CAC​TGG​CGT​CTT​CA-3′; human GAPDH-Reverse, 5′-GTC​ATG​AGT​CCT​TCC​ACG​ATA​CC-3′.

### 2.3 Correlation and *INHBA*-Related Gene Enrichment Analysis

Functional networks and gene connectivity data were extracted using the STRING database (https://www.string-db.org/, version 11.5). The parameters used were as follows: minimum required interaction score [“medium confidence (0.400)”], meaning of network edges (“evidence”), max number of interactors to show (“no more than 10 interactors” in first shell), and active interaction sources (“all”).

The correlation between expression levels of *INHBA* and other mRNAs in cervical cancer was determined using TCGA data, and the Pearson correlation coefficient was calculated for all correlation analyses. The top100 genes most significantly positively correlated with *INHBA* were selected for enrichment analysis to reflect their functional roles. Gene Ontology (GO) term enrichment analysis was performed using the R package clusterProfiler (v3.14.3). For KEGG pathway analysis, the R package clusterProfiler (v3.14.3) was employed. GSEA between high- and low-*INHBA* groups was carried out using the R package clusterProfiler (version 3.14.3). The process was repeated 1,000 times for each analysis and c2.cp.v7.2.symbols.gmt in MSigDB Collections were used as a reference gene collection. For gene sets to be considered significantly enriched, the false discovery rate (FDR) q-value needed to be smaller than 0.25 and P adjust < 0.05.

### 2.4 Survival Prognosis Analysis

We obtained patient survival data from TCGA and performed survival analysis using R packages, including package survival (version 3.2.10) and survminer (version 0.4.9). We selected 50% as the threshold, and the cohorts were divided into low-and high-expression groups. The relationships between *INHBA* expression and patient prognosis, including Overall Survival (OS), disease Specific Survival (DSS), and Progression Free Interval (PFI), were investigated.

### 2.5 Immune Cell Infiltration Analysis

Immune infiltration analysis of cervical cancer was performed using the single-sample gene set enrichment analysis (ssGSEA) method with the R package GSVA (version 1.34.0). To explore the correlation between *INHBA* and the infiltration levels of immune cells and the association of immune cell infiltration with the different expression groups of *INHBA*, Spearman’s rank correlation and Wilcoxon rank sum test were used. Statistical significance was set at *p* < 0.05.

## 3 Results

### 3.1 *INHBA* Expression Analysis in Pan-Cancer and Cervical Cancer Cell Lines

**FIGURE 1:**
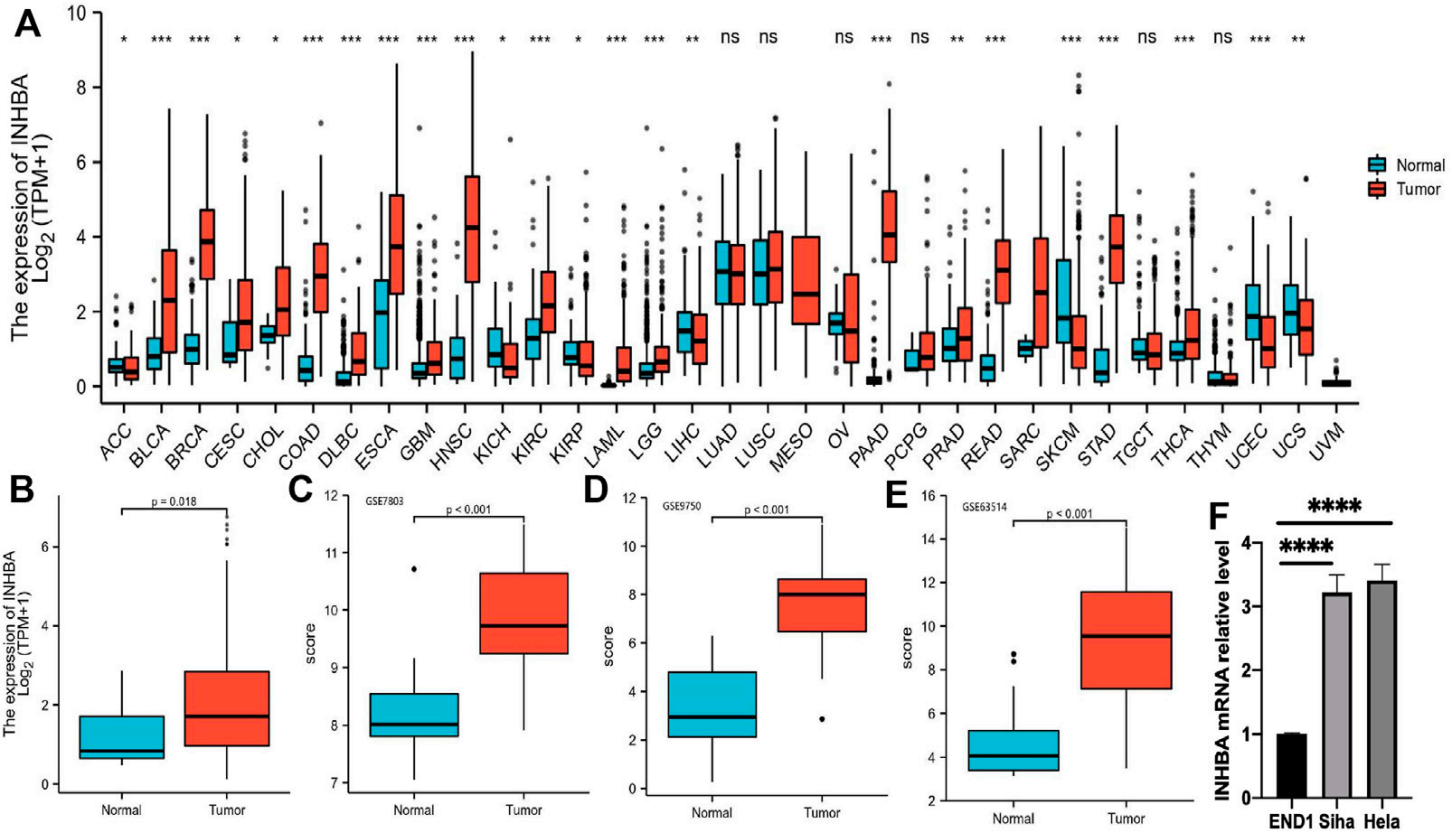
*INHBA* expression level analysis in pan-cancer. **(A)** *INHBA* expression in normal and tumor tissues in TCGA and GTEx data. **(B)** Boxplot representation of *INHBA* expression in cervical cancer tissue. **(C–E)** *INHBA* expression in normal cervical tissues and cervical cancer epithelial component from GSE7803, GSE9750 and GSE63514. **(F)** qRT-PCR analysis of *INHBA* expression in the indicated cell lines.

We performed pan-cancer analyses using the Mann-Whitney U test (Wilcoxon rank sum test) to compare *INHBA* expression in normal tissues and tumor samples using RNA sequencing data obtained from TCGA and GTEx databases (Figure 1A). *INHBA* expression was significantly higher in 19 tumor types, including BLCA (P < 0.001), BRCA (P < 0.001), CESC (*p* = 0.018), CHOL (*p* = 0.049), COAD (P < 0.001), DLBC (P < 0.001), ESCA (P < 0.001), GBM (P < 0.001), HNSC (P < 0.001), KIRC (P < 0.001), LAML (P < 0.001), LGG (P < 0.001), PAAD (P < 0.001), PAAD (P < 0.001), PRAD (*p* = 0.004), READ (P < 0.001), STAD (P < 0.001), and THCA (P < 0.001). In contrast, *INHBA* was expressed at low levels in the ACC (*p* = 0.015), KICH (*p* = 0.02), KIRP (*p* = 0.049), LIHC (*p* = 0.002), SKCM (P < 0.001), UCEC (P < 0.001), and UCS (*p* = 0.007). Furthermore, there were no significant differences in LUAD, LUSC, OV, PCPG, THYM, and TGCT (P > 0.05). We selected CESC for further boxplot presentation and confirmed that *INHBA* expression was upregulated in CESC tumor samples compared to that in normal tissues (*p* = 0.018) (Figure 1B). In addition, *INHBA* was upregulated in cervical cancer in GSE7803, GSE9750, and GSE63514 datasets (Figures 1C–E). To further confirm our observations, we examined *INHBA* expression in cervical cancer lines, including SiHa and HeLa, compared to that in the normal epithelial cell line, END1, using qRT-PCR. The results of the qRT-PCR analysis demonstrated that *INHBA* mRNA expression was higher in cervical cancer cell lines than in normal cervical epithelial cell lines (Figure 1F).

### 3.2 Correlation and *INHBA*-Related Gene Enrichment Analysis

**FIGURE 2:**
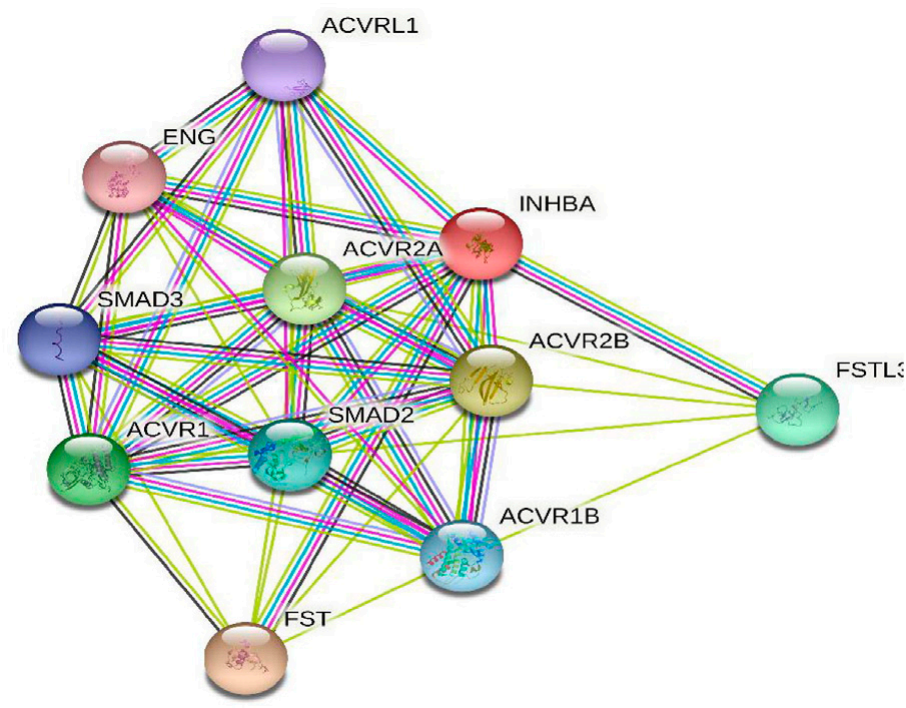
Top 10 *INHBA*-binding proteins obtained by STRING database.

**FIGURE 3:**
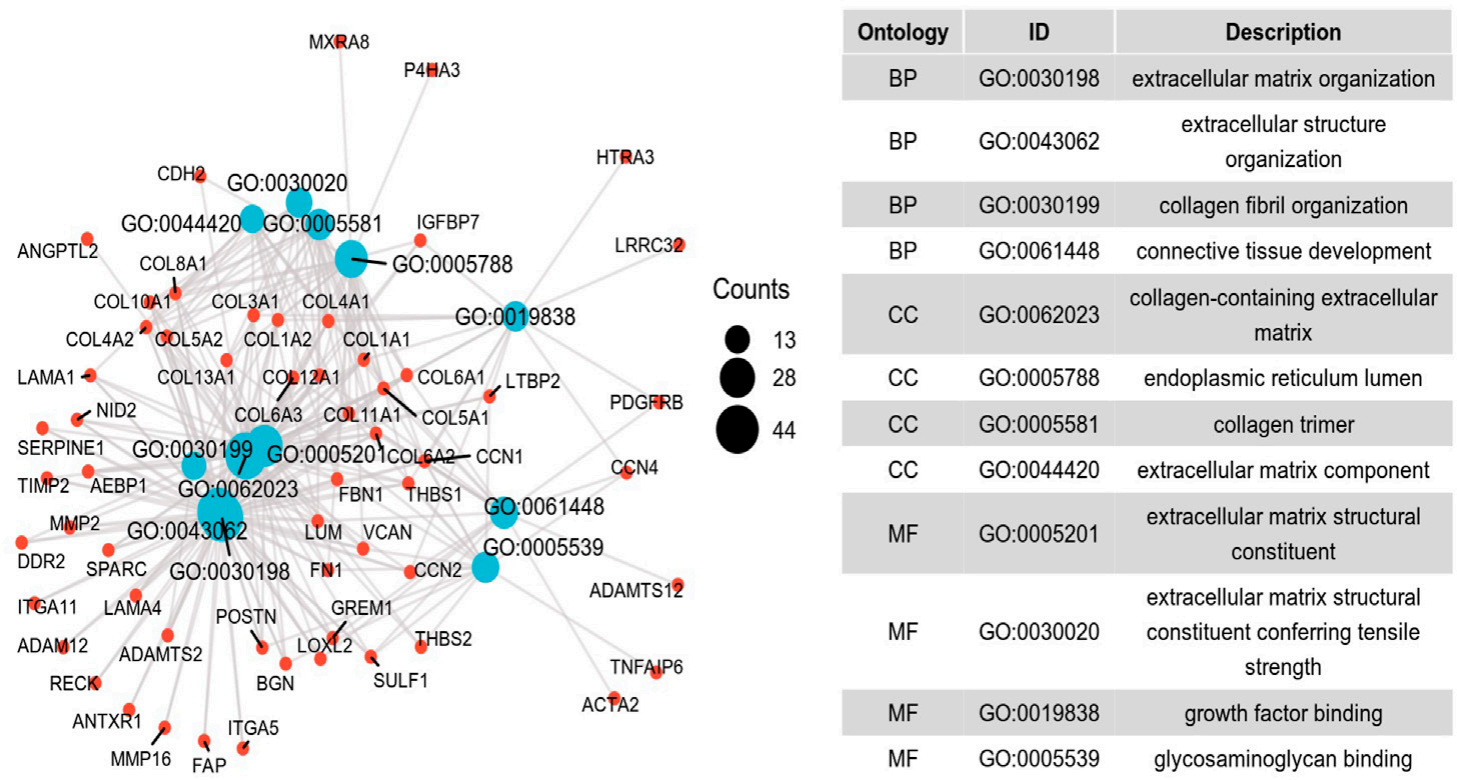
Significant Gene Ontology terms of the top 100 genes most positively associated with *INHBA*, including BP (biological processes), MF (molecular function) and CC (cell component).

**FIGURE 4:**
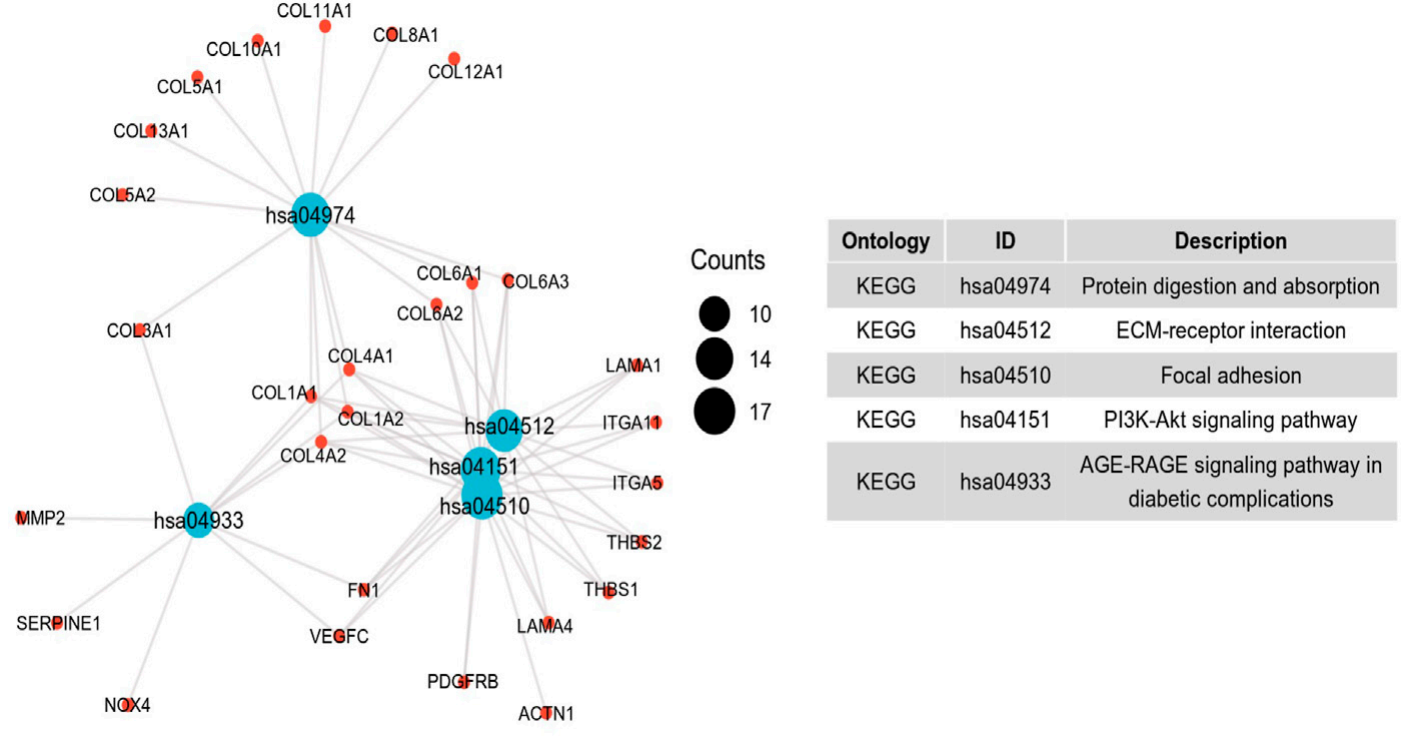
Significant KEGG pathway of the top 100 genes most positively associated with *INHBA*.

**FIGURE 5:**
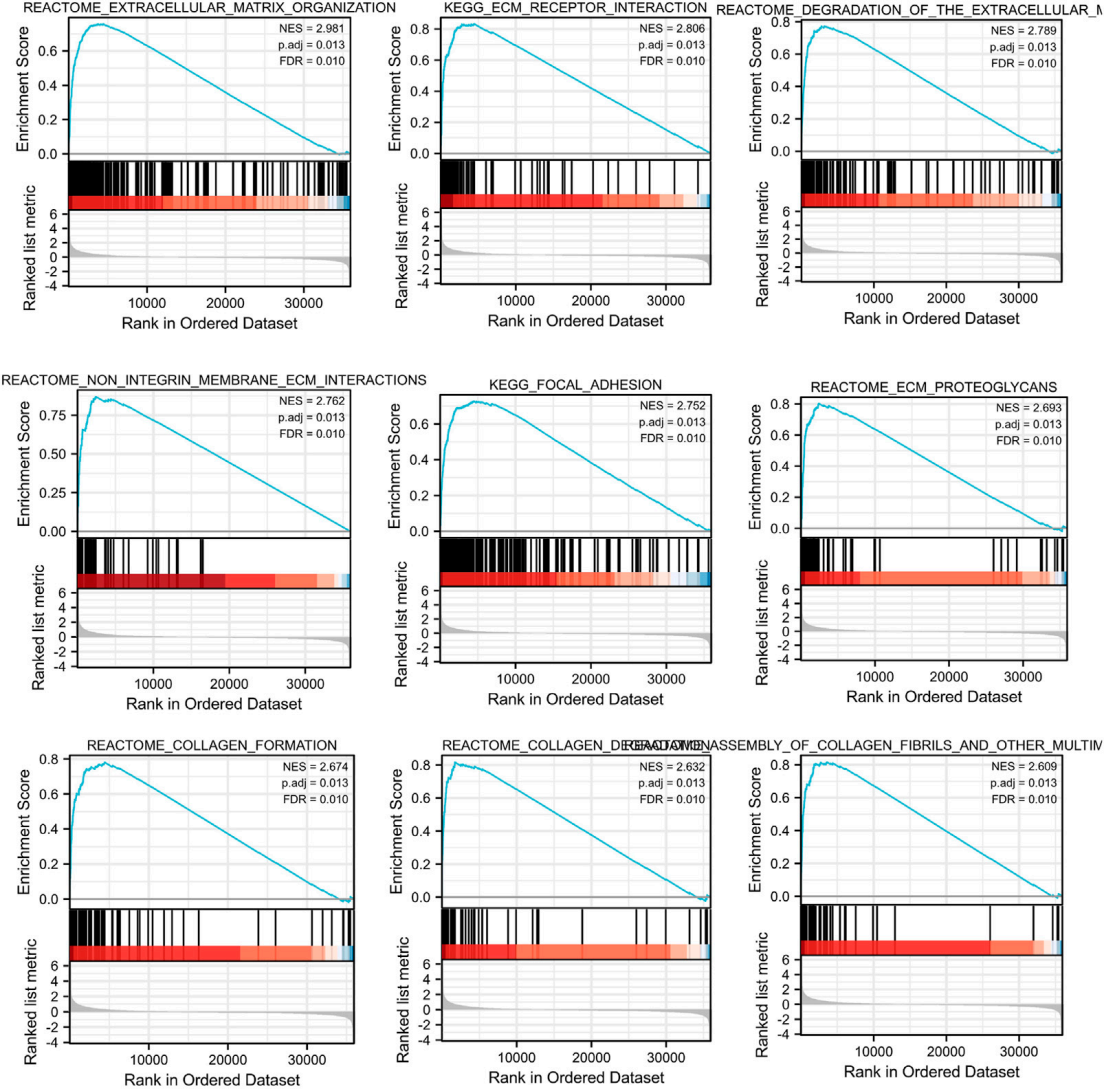
Top 9 significant results most positively associated with *INHBA*, including Reactome pathways and KEGG pathways.

To further investigate the potential molecular mechanism of *INHBA* involvement in cervical cancer development and progression, the STRING tool was utilized. Based on the STRING tool, a database of known and predicted protein-protein interactions, we obtained the top 10 *INHBA* -binding proteins. Figure 2 demonstrates the interaction network of these proteins. We downloaded the expression data from the TCGA cancer browser website to investigate the functional enrichment information and pathways involved. Next, we selected the top100 genes that were most positively correlated with *INHBA* using the R stats package (Supplementary Table S1). As shown in Figure 3, the GO enrichment analysis of related genes revealed a significant enrichment of GO terms associated with extracellular matrix, such as extracellular matrix organization, collagen-containing extracellular matrix, extracellular matrix structural constituent, and others. The KEGG data analysis indicated that the “PI3K-Akt signaling pathway” might be related to the involvement of *INHBA* in tumor pathogenesis (Figure 4). Further, we performed GSEA to identify *INHBA* -related signaling pathways, and a total of 1069 pathways were enriched. There were 536 datasets that showed significant differential enrichment in the *INHBA* high expression phenotype. We selected the top nine datasets with a high normalized enrichment score (NES) (Figure 5). The results revealed that ECM receptor interaction and focal adhesion were significantly enriched in the KEGG pathway. In addition, extracellular matrix organization, degradation of the extracellular matrix, NON-integrin membrane ECM interactions, ECM proteoglycans, collagen formation, and collagen degradation were significantly enriched in Reactome pathway analysis (Supplementary Table S2).

### 3.3 Association Between *INHBA* Expression and Cancer Patients Survival Prognosis

**FIGURE 6:**
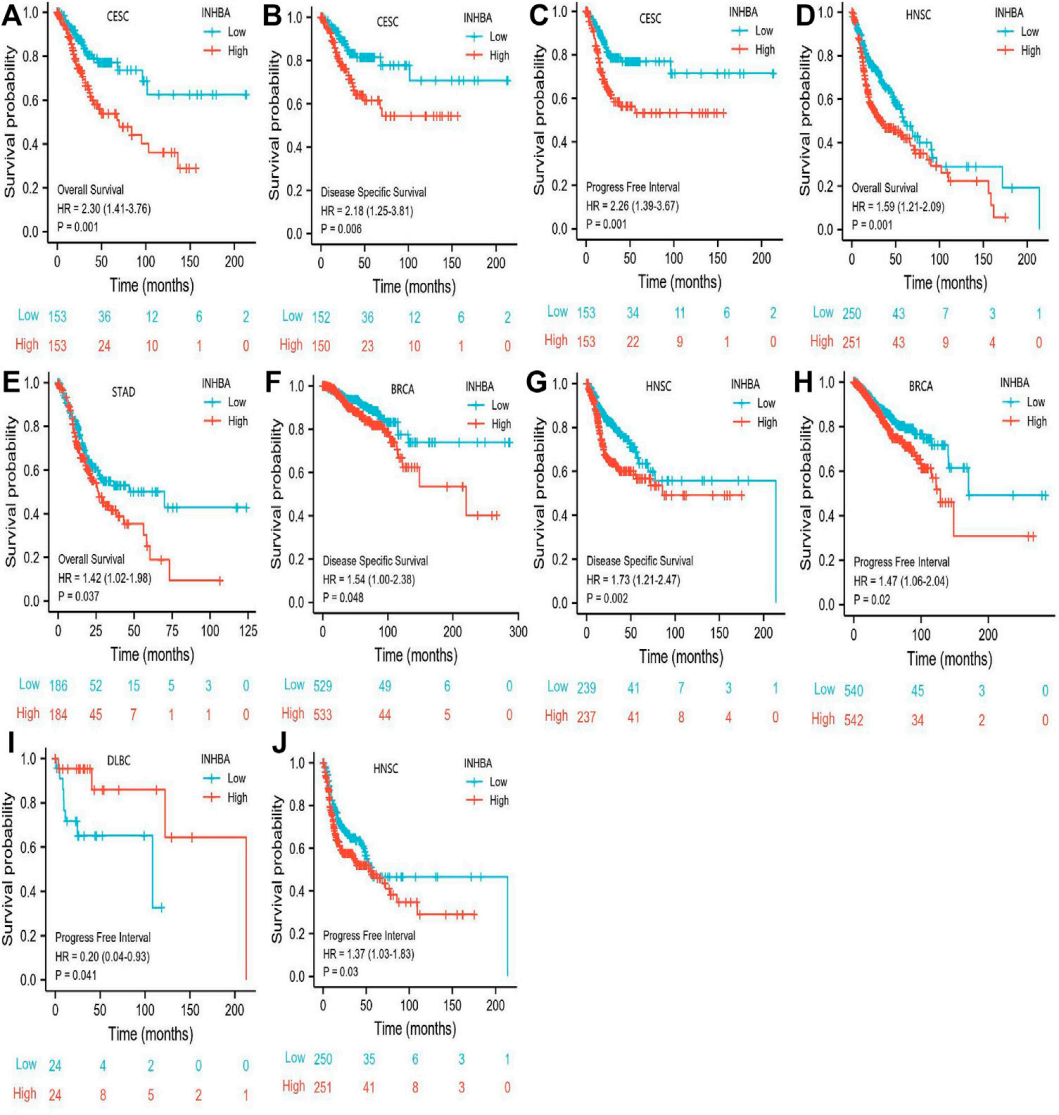
Association between *INHBA* expression and cancer survival prognosis. **(A)** CESC *INHBA* OS Survival. **(B)** CESC *INHBA* DSS Survival. **(C)** CESC *INHBA* PFI Survival. **(D)** HNSC *INHBA* OS Survival. **(E)** STAD *INHBA* OS Survival. **(F)** BRCA *INHBA* DSS Survival. **(G)** HNSC *INHBA* DSS Survival. **(H)** BRCA *INHBA* PFI Survival. **(I)** DLBC *INHBA* PFI Survival. **(J)** HNSC *INHBA* PFI Survival.

The characteristics of cervical cancer patients are listed in Table 1, in which clinical data and mRNA expression profiles from 306 patients with cervical cancer were collected from the TCGA database. Mean *INHBA* expression was defined as the cutoff to classify patients into high expression (*n* = 153) and low expression groups (*n* = 153). To identify the prognostic value of markers for cervical cancer, we evaluated the correlation between high expression of *INHBA* and patients’ survival time using Kaplan-Meier analysis with Cox regression, including OS, DSS, and PFI. The results demonstrated that higher *INHBA* expression was associated with poorer prognosis for OS (*p* = 0.001, HR = 2.30, 95% CI: 1.41–3.76) (Figure 6A), DSS (*p* = 0.006, HR = 2.18, 95% CI: 1.25–3.81) (Figure 6B), and PFI (*p* = 0.001, HR = 2.26, 95% CI: 1.39–3.67) (Figure 6C) in cervical cancer. Furthermore, we placed *INHBA* in the broader context of cancer by performing several pan-cancer analyses. We found that higher *INHBA* expression was associated with poorer OS in patients with HNSC (*p* = 0.001, HR = 1.59, 95% CI: 1.21–2.09) (Figure 6D) and STAD (*p* = 0.031, HR = 1.42, 95% CI: 1.02–1.98) (Figure 6E). There was a trend toward decreased DSS with increasing *INHBA* expression in BRCA (*p* = 0.048, HR = 1.54, 95% CI: 1.00–2.38) (Figure 6F) and HNSC (*p* = 0.002, HR = 1.73, 95% CI: 1.21–2.47) (Figure 6G). Moreover, upon analysis of the PFI data of patients with BRCA (*p* = 0.02, HR = 1.47, 95% CI: 1.06–2.04) (Figure 6H), DLBC (*p* = 0.041, HR = 0.2, 95% CI: 0.04–0.93) (Figure 6I), and HNSC (*p* = 0.03, HR = 1.37, 95% CI: 1.03–1.83) (Figure 6J), high *INHBA* expression was found to be correlated with poor prognosis.

**TABLE 1:** Characteristics of cervical cancer patients based on TCGA database.

Characteristic	Low expression of *INHBA*	High expression of *INHBA*
n	153	153
T stage, n (%)		
T1	70 (28.8%)	70 (28.8%)
T2	41 (16.9%)	31 (12.8%)
T3	10 (4.1%)	11 (4.5%)
T4	3 (1.2%)	7 (2.9%)
N stage, n (%)		
N0	77 (39.5%)	57 (29.2%)
N1	21 (10.8%)	40 (20.5%)
M stage, n (%)		
M0	59 (46.5%)	57 (44.9%)
M1	7 (5.5%)	4 (3.1%)
Clinical stage, n (%)		
Stage I	79 (26.4%)	83 (27.8%)
Stage II	38 (12.7%)	31 (10.4%)
Stage III	23 (7.7%)	23 (7.7%)
Stage IV	9 (3%)	13 (4.3%)
Radiation therapy, n (%)		
No	56 (18.3%)	66 (21.6%)
Yes	97 (31.7%)	87 (28.4%)
Primary therapy outcome, n (%)		
PD	8 (3.7%)	15 (6.8%)
SD	4 (1.8%)	2 (0.9%)
PR	4 (1.8%)	4 (1.8%)
CR	104 (47.5%)	78 (35.6%)
Race, n (%)		
Asian	11 (4.2%)	9 (3.4%)
Black or African American	15 (5.7%)	16 (6.1%)
White	108 (41.4%)	102 (39.1%)
Histological type, n (%)		
Adenosquamous	29 (9.5%)	24 (7.8%)
Squamous cell carcinoma	124 (40.5%)	129 (42.2%)
Histologic grade, n (%)		
G1	10 (3.6%)	9 (3.3%)
G2	72 (26.3%)	63 (23%)
G3	59 (21.5%)	60 (21.9%)
G4	0 (0%)	1 (0.4%)
Age, meidan (IQR)	46 (39, 60)	47 (38, 54)

### 3.4 The Correlation Between *INHBA* Expression and Immune Cell Infiltration

**FIGURE 7:**
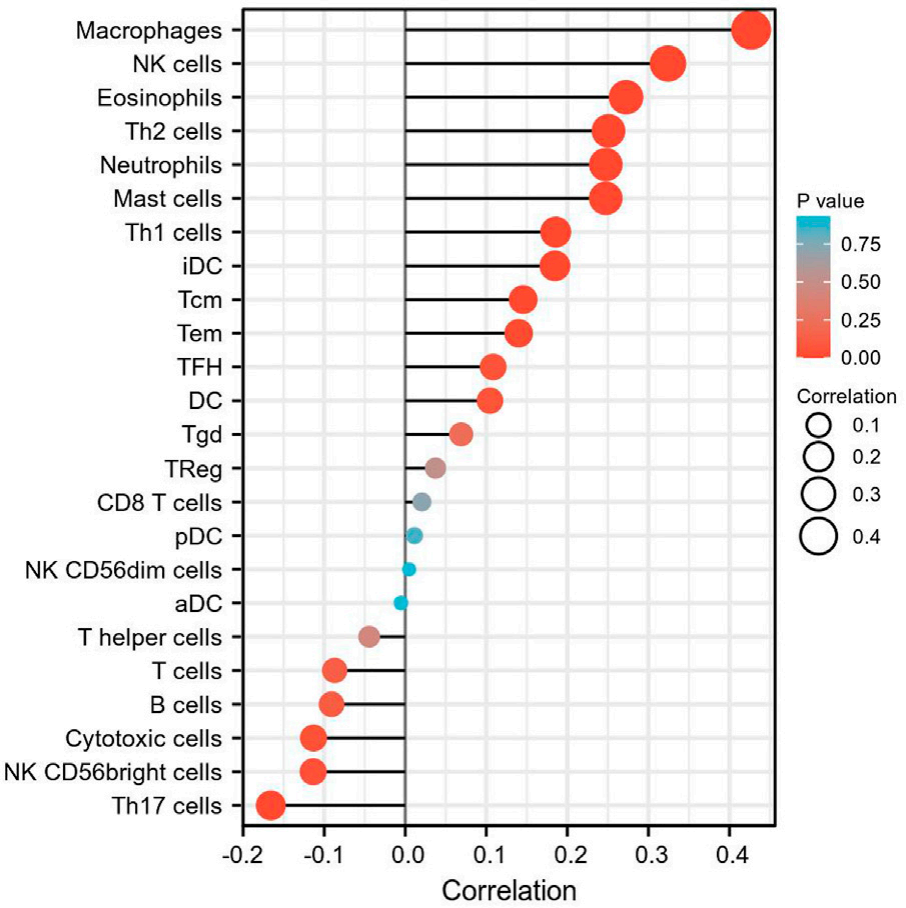
The correlation between *INHBA* expression level and 24 immune cell types. The size of dots indicate the absolute value of Spearman r.

**FIGURE 8:**
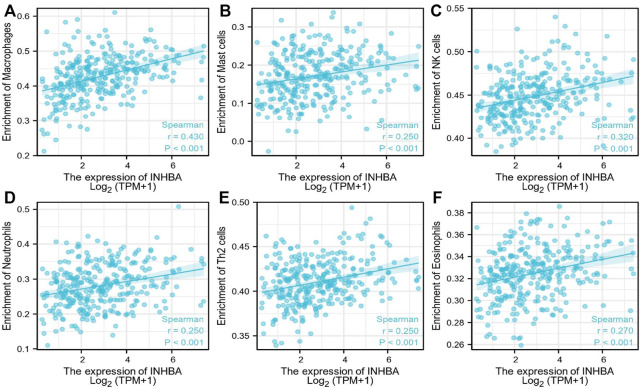
Immune cells postively correlated with *INHBA* expression. **(A)** Correlation between *INHBA* expression and Macrophages. **(B)** Correlation between *INHBA* expression and Mast cells. **(C)** Correlation between *INHBA* expression and NK cells. **(D)** Correlation between *INHBA* expression and Neutrophils. **(E)** Correlation between *INHBA* expression and Th2 cells. **(F)** Correlation between *INHBA* expression and Eosinophils.

ssGSEA with Spearman’s rank correlation was employed to measure the correlation between *INHBA* expression and infiltration levels of 24 immune cell types (Figure 7). The results showed that *INHBA* expression was correlated with several subsets of myeloid cells, including macrophages and mast cells, natural killer (NK) cells, neutrophils, Th2 cells, and eosinophils. Our study indicated that the expression of *INHBA* was positively associated with these immune cell types (Figures 8A–F, P < 0.001).

## 4 Disscusion

Despite advances in screening, diagnosis, and treatment, cervical cancer remains a leading cause of cancer-related deaths worldwide. Therefore, accurate biomarkers are needed for the early detection and monitoring of disease progression. Previous studies have proven that *INHBA* is significantly increased in various tumors and it may be a potential prognostic biomarker for predicting the survival outcome of cancer patients Lyu et al. (2018); Li et al., 2020. While the role of *INHBA* has been comprehensively studied in many cancer types, its function in cervical cancer remains insufficiently understood. In this study, we attempted to address the role of *INHBA* in cervical cancer for the first time.

*INHBA* encodes a subunit of activin and inhibin, members of the TGF*β* superfamily, which play context-dependent roles in cancer progression Li et al., 2020. Previous studies have shown that *INHBA* expression is upregulated in lung cancer and esophageal adenocarcinoma and has been shown to promote cell proliferation Liang et al. (2012); Dai et al. (2018). Furthermore, upregulated *INHBA* has also been implicated in promoting cancer stem cell and metastatic properties Hadadi et al. (2020). We also found that *INHBA* was highly overexpressed in most cancer types, including cervical cancer tissues, compared to that in adjacent normal tissues. In our study, we found that high expression of *INHBA* correlated with poor OS, DSS, and PFI in cervical cancer patients. Identical results were also observed at the pan-cancer level. High expression of the protein in tumors was associated with significantly shorter survival rates than that of patients whose tumors expressed lower levels of *INHBA* in BRCA, DLBC, HNSC, and STAD. Based on these findings, *INHBA* can be considered as a potential therapeutic target. Kalli et al. demonstrated that knocking down *INHBA* levels delayed primary breast tumor growth and suppressed the formation of lung metastases *in vivo* Kalli et al. (2019).

However, in some malignancies, *INHBA* serves as a tumor suppressor gene. Since *INHBA*/activins proteins are multifunctional ligands and their superfamily member, TGF-*β*, is closely involved in angiogenesis, *INHBA* may also play a role in tumor angiogenesis. For example, *INHBA* substantially inhibits tumor angiogenesis in gastric cancer *in vivo* Kaneda et al. (2011) and neuroblastoma models Liang et al. (2012). To the best of our knowledge, inhibition of angiogenesis potentially prevents tumor growth and metastasis to other organs.

To further investigate the function of *INHBA* in detail, we performed functional annotation based on the enrichment analysis. Among the first ten top-ranked primary *INHBA* interactors in the STRING protein-protein interaction (PPI) network, most genes were involved in the TGF-*β* signaling pathway. Previous studies have also demonstrated that *INHBA* plays a role in the regulation of cancer cell growth, proliferation, and survival *via* the TGF-*β* signaling pathway Chen et al. (2019); Yu et al. (2021); Basu et al. (2015). GO and GSEA analysis revealed that *INHBA* -related genes were associated with extracellular matrix, collagen formation and degradation, the misregulation of which is a key factor in epithelial to mesenchymal transition (EMT) Burke et al. (2015). Basu et al. Basu et al. (2015) also indicated that *INHBA* expression contributes to EMT in both normal and ovarian cancer cells. Several studies have shown that *INHBA* controls cell proliferation and apoptosis through the PI3K/Akt pathway Tsai et al. (2019); Loomans and Andl (2014), which is consistent with our KEGG pathway analysis.

The tumor microenvironment is a complex assembly of tumor, immune, stromal, and extracellular components, and it has emerged as an important component that contributes to tumor progression and metastasis Lee et al. (2019). Tumor-associated macrophages are considered essential components of the tumor microenvironment and play critical roles in the regulation of tumor progression Zhu et al. (2017). According to our research, there was a significant positive correlation between *INHBA* expression and macrophage infiltration. Additionally, *INHBA* has been shown to affect macrophage polarization *in vitro* Hreha et al. (2020). We hypothesized that *INHBA* may influence the tumor microenvironment by regulating macrophage polarization, which in turn affects tumor cells. Based on our research, mast cells, NK cells, neutrophils, Th2 cells, and eosinophils were also positively correlated with *INHBA* expression. However, the role of *INHBA* in the immune system is not fully understood, with only a handful of publications providing evidence for its involvement in T cell biology and neutrophils Locci et al. (2016); Sideras et al. (2013). Thus, a more comprehensive survey of the relationship between *INHBA* and immune infiltration is warranted.

In conclusion, we found that *INHBA* was overexpressed in cervical cancer and was significantly related to poor prognosis. *INHBA* may be involved in tumor progression and metastasis. Moreover, all the results indicated that *INHBA* is likely to play a key role in macrophage polarization and immune cell infiltration. Therefore, it could be used as a potential prognostic target for cervical cancer.

Although our study has uncovered some new facts, it also has the following limitations. First, our study is based on bioinformatics analyses, and the data are from public databases; therefore, it lacks the verification of clinical data of our hospital. Second, further validation studies with a long-term follow-up and larger cohorts of patients are needed to definitively validate *INHBA* as a prognostic predictor. Finally, further in-depth studies are required to address the relationship between *INHBA* gene expression and immune infiltration in more detail.

## Data Availability

Publicly available datasets were analyzed in this study. This data can be found here: TCGA data portal (https://portal.gdc.cancer.gov/), UCSC Xena (https://xenabrowser.net/datapages), and GEO database (https://www.ncbi.nlm.nih.gov/geo/).

## Glossary

BLCA: Bladder urothelial carcinoma
BRCA: Breast invasive carcinoma
CESC: Cervical squamous cell carcinoma and endocervical adenocarcinoma
CHOL: Cholangiocarcinoma
COAD: Colon adenocarcinoma
DLBC: Lymphoid Neoplasm Diffuse Large B-cell Lymphoma
DSS: disease Specific Survival
ESCA: Esophageal carcinoma; GBM: Glioblastoma multiforme
GEO: Gene Expression Omnibus
GSEA: gene-set enrichment analysis
GO: Gene Ontology
KEGG: Kyoto Encyclopedia of genes and genomes
HNSC: Head and neck squamous cell carcinoma
*INHBA*: Inhibin A
LAML: Acute myeloid leukemia
LGG: Lower grade glioma
LIHC: Lever hepatocellular carcinoma
LUAD: Lung adenocarcinoma
LUSC: Lung squamous cell carcinoma
KICH: Kidney chromophobe
KIRC: Kidney renal clear cell carcinoma
KIRP: Kidney renal papillary cell carcinoma
OS: Overall Survival
OV: Ovarian serous cystadenocarcinoma
PAAD: Pancreatic adenocarcinoma
PCPG: Pheochromocytoma and paraganglioma
PFI: Progress Free Interval
PRAD: Prostate adenocarcinoma
READ: Rectum adenocarcinoma
SKCM: Skin cutaneous melanoma
STAD: Stomach adenocarcinoma
TCGA: The Cancer Genome Atlas
TGCT: Testicular germ cell tumor
THCA: Thyroid carcinoma
THYM: Thymoma
UCEC: Uterine corpus endometrial carcinoma
UCS: Uterine carcinosarcoma
